# Professional Care Experiences of Persons With Suicidal Ideation and Behavior: Model Development Based on a Qualitative Meta-Synthesis

**DOI:** 10.2196/27676

**Published:** 2021-10-28

**Authors:** Mareike Hechinger, André Fringer

**Affiliations:** 1 Institute of Nursing School of Health Professions Zurich University of Applied Sciences Winterthur Switzerland

**Keywords:** nursing care, health care professionals, suicidal behavior, suicidal inclinations, suicidal ideation, inpatient, outpatient, eHealth, mHealth, mental health, suicide, stress

## Abstract

**Background:**

Health care professionals (HCPs) are challenged in caring for persons with suicidal ideation or behavior. For affected persons, professional care is essential, and being interviewed about their experiences can be stressful. The experiences of persons ideating or attempting suicide are essential to designing eHealth products to support them in crises and provide continuous care.

**Objective:**

This study aimed to synthesize published qualitative research about how persons with suicidal thoughts or behavior experience inpatient or outpatient care. A model will be derived from the meta-synthesis to guide HCPs in their work with affected persons and provide a thorough needs assessment for eHealth development.

**Methods:**

A qualitative meta-synthesis was conducted using an inductive approach, as proposed by Sandelowski and Barroso. The inclusion criteria were studies in English and German that dealt with persons who ideated or attempted suicide. Relevant articles were identified by searching the PubMed and Cinahl databases and by hand searching relevant journals and reference lists. The findings of each study were analyzed using initial and axial coding, followed by selective coding. Finally, a conceptual model was derived.

**Results:**

In total, 3170 articles were identified in the systematic literature search. Articles were screened independently by 2 researchers based on the eligibility criteria. Finally, 12 studies were included. The central phenomenon observed among persons ideating or attempting suicide is their process from feeling unanchored to feeling anchored in life again. During inpatient and outpatient care, they experience being dependent on the skills and attitudes of HCPs. While helpful skills and attitudes support persons ideating or attempting suicide to reach their feeling of being anchored in life again, adverse interactions are experienced negatively and might lead to prolonging or maintaining the feeling of being unanchored in life.

**Conclusions:**

The study promotes a differentiated view of the experiences of persons ideating or attempting suicide. The derived conceptual model can guide HCPs in their work with affected persons to support affected persons during their recovery. Moreover, the conceptual model is useable as a springboard to develop eHealth solutions for crisis situations and long-term care.

## Introduction

Since 2000, the suicide rate worldwide has decreased. Nevertheless, in 2016, nearly 800,000 deaths were registered due to suicide, with a mortality rate of 10.6 per 100,000 inhabitants. While women attempt suicide 2 to 4 times more often than men, there are nearly 2 male suicide deaths for 1 female death [1]. However, Klonsky et al [2] state difficulties in suicide research, especially in statistics, due to the stigma associated with suicide, leading to underreported suicide rates.

The term suicide describes a “death caused by self-directed injurious behavior with an intent to die as a result of the behavior” [3]. A suicide attempt comprises self-directed, potentially injurious behavior. The suicide attempt is performed with an intent to die, though the attempt itself is nonfatal. The terms suicidal self-directed violence and suicidal behavior are used interchangeably. Thinking, considering, or planning to attempt suicide is called suicidal ideation [3].

The consequences of suicidal ideation and behavior are complex. On a personal level, people who are ideating or attempting suicide experience a crisis. Persons who attempted suicide also have difficulties coping with stigma in their interpersonal relationships [4]. For bereaved family members, it is a challenge to cope with grief and guilt. They fear social stigmatization [5]. For nurses, the consequences mainly include unfavorable attitudes towards persons who attempted suicide on the ward, as a survey indicates [6]. Nurses who experience a suicide or suicide attempt at work indicate that such an experience can lead to shock, anger, and frustration. They highlight the importance of being supported afterward [7]. Therapists describe the challenge in the limited time for direct contact with affected persons while prioritizing standardized assessments for diagnostics instead of therapeutic conversations [8]. Emergency department physicians are concerned about overlooking a serious suicide risk [9]. Studies indicate a need for increased knowledge, training, and clinical skills among physicians [9,10].

On a broader level, suicide causes high costs for society, as studies in Spain, Ireland, and Australia indicate. They recommend increased efforts for suicide prevention and health education [11-13]. Over the last few years, the internet has become another option for suicide prevention. Different applications and strategies have been developed and tested [14,15]. The COVID-19 pandemic is challenging people around the world, especially those already struggling with mental illness. The pandemic has also challenged previously established mental health services. Service providers had to find new ways to maintain physical distance while providing treatment and care [16]. Internet-based interventions were highlighted as a convenient option during the pandemic due to actual challenges [17]. Mental health providers perforce offered telemental services [16]. Although there are evidence-based strategies to prevent suicide, difficulties in realization remain prevalent [18]. Especially for telemedicine approaches, challenges arise due to missing legal regulation and concerning ethical aspects in acute situations with suicide risk. For this reason, family and social networks should be involved [16,17]. Difficulties arise during the call or video session, especially due to the patient's remote location [16,19]. In the context of adolescents, Holland et al [19] recommend confirming the physical location and determining if an adult is present. In their review, they conclude that risk assessment and safety planning via telehealth are safe and effective. Studies examining internet-based interventions for persons at risk of having suicidal thoughts or behavior have ethical and practical barriers. Nevertheless, the opinions and experiences of persons with suicidal thoughts or behavior are crucial for developing and testing internet-based applications [20].

These studies show the need for support of health care professionals (HCPs) in their work with persons who ideate or attempt suicide and for the development of evidence-based eHealth interventions derived from the experiences of persons in a suicidal crisis. Therefore, we decided to synthesize the existing qualitative research about how persons with suicidal thoughts or behavior experience inpatient or outpatient care. Our study sought to assess the experiences persons with suicidal thoughts or behavior have had with the professional care they receive in an inpatient or outpatient setting. The aim is to derive a conceptual model to guide HCPs in working with affected persons and provide support during their recovery. Furthermore, the model will be reviewed critically with current literature in the context of health services and eHealth usage of affected persons, from which conclusions will be drawn for the development and revision of eHealth applications. HCP refers to nurses with or without mental health or psychiatric specialization, psychologists, and psychiatrists.

## Methods

### Overview

A qualitative meta-synthesis was conducted using the approach by Sandelowski and Barroso [21] and followed the recommended six steps: (1) formulating a purpose, (2) systematically searching for and retrieving qualitative research reports, (3) appraising the research reports, (4) classifying the findings, (5) conducting a meta-summary, and (6) developing a meta-synthesis. The meta-synthesis method enables an interpretive integration of the results of all included qualitative studies, basing the results on a larger sample than would be possible from one single qualitative research study. Consequently, coherent experiences and events of the research topic can be described and explained instead of merely summarizing them [21]. Therefore, the purpose of this article was to conduct a meta-synthesis of experiences by persons with suicidal thoughts or behavior with nursing care.

### Searching for and Appraising the Research Reports

The systematic literature search was performed between July and October 2016, with an update in June 2021, in PubMed, Cinahl, Medline-OVID, Embase-OVID, Psyndex-OVID, and PsycINFO-OVID. The search terms were (experience OR “lived experience” OR attitudes OR “patient perspective” OR perception) AND (“qualitative research” OR qualitative OR “qualitative design” OR “qualitative study” OR “phenomenological study” OR phenomenology OR “grounded theory”) AND (“suicidal ideation” OR suicidal OR suicide OR “suicidal patient” OR “suicidal behavior” OR “suicidal behaviour“) AND (nursing OR nurses OR hospitalization OR “inpatient care” OR outpatient). Search terms were slightly adjusted to fit the different search systems; in Cinahl, subject headings were used, and in PubMed, medical subject headings were used. Since we aimed for a sensitive search strategy, an additional manual search was conducted in 8 specialist journals (ie, Zeitschrift für Psychologie, The Journal of Crisis Intervention and Suicide Prevention, European Journal of Psychological Assessment, GeroPsych, Nordic Psychology, PRAXIS, Zeitschrift für Kinder-und Jugendpsychiatrie und Psychotherapie, and European Psychologist). Afterward, a literature search of the reference lists of the included studies was conducted.

We included studies with a qualitative study design, reported experiences of persons with suicidal thoughts or behavior regarding nursing care, and were published in English or German. Studies that reported experiences of persons with self-injuring tendencies without suicidal self-directed violence were excluded. We also excluded studies that focused solely on the experiences of family members or HCPs. Studies were excluded if a theory-based or deductive qualitative approach was used. Records were independently screened and identified for eligibility by 2 independent researchers. Discrepancies were discussed with a third researcher. Based on the title and abstract, 2 researchers read the full texts of the studies that appeared to meet the inclusion criteria. Next, the included studies were appraised using the checklist for qualitative research of the critical appraisal skills program (CASP) [22]. The CASP checklist is a useful tool for appraising qualitative studies and systematically identifying the strengths and weaknesses of the assessed studies in design and analysis [23]. The tool appraises the quality of studies but not the quality of the appraisal itself. It consists of 10 “yes” or “no,” and 2 researchers can select “can’t tell” as needed. The results were compared and discussed. The remaining discrepancies were clarified with a third researcher. We defined criteria for each point and a cut-off score that resulted in studies being excluded when they had less than seven “yes” points in the 10-point questionnaire.

### Classifying the Findings, Conducting a Meta-Summary, and Developing a Meta-Synthesis

When conducting a qualitative meta-synthesis, the findings of studies should be read to assess which methods were applied and how the data were interpreted. Therefore, the included studies were classified with a recommended typology [21]. Studies were categorized as thematic surveys [24,25], conceptual or thematic descriptions [26-28], or interpretive explanations [29-35].

The basic assumption for conducting a meta-summary and developing a meta-synthesis was that the results of the included studies are interpretations of the data collected by the researchers. Consequently, the results sections of the included studies were treated as transcripts of a qualitative study and used as meta-synthesis data. The results sections of the studies were read several times and then analyzed inductively [36]. MAXQDA2018 (2018; VERBI GmbH) was used to support and manage the analysis process. As a first cycle method, initial sentence by sentence coding of the published material was used to go beyond what was said and discover deeper patterns [36]. In the second step, the results sections were then axially coded. With the method of constant comparison, similarities and differences could be identified. Both helped to identify categories and subcategories. Axial coding promoted a meta-summary reflecting the contents of the included studies. We used constant and comparison and interwove the emerging categories inductively to develop generic categories. A deeper theoretical level of abstraction could be gained with selective coding as the third cycle method and further axial coding [36]. With this interpretive approach, the main concepts emerged from the data, and a conceptual model was derived.

## Results

### Overview

**Figure 1:**
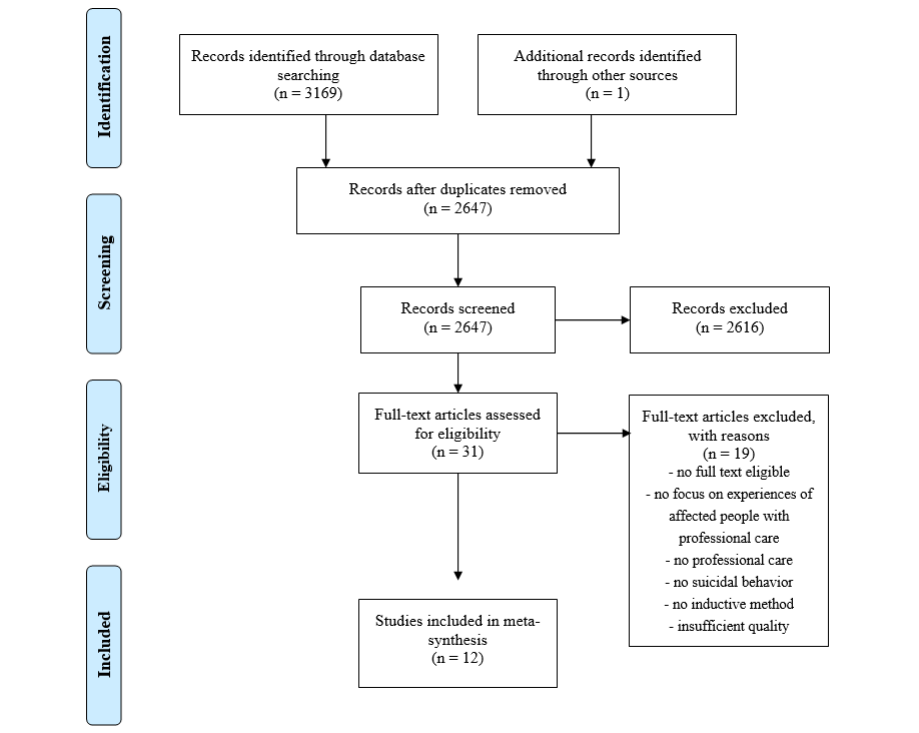
Flow diagram of the review process.

A total of 3169 studies were identified based on a systematic literature search of databases. One additional study could be identified by a hand search in relevant journals or by screening the references of the included articles. The flowchart of the literature search is shown in Figure 1. Finally, 12 qualitative studies with a total of 208 persons were included in the meta-synthesis, including affected persons (n=176), parents (n=5) or other family members (n=2), nurses or psychiatric nurses (n=27), and psychiatrists (n=3). Affected persons received outpatient or inpatient care due to suicidal ideation or suicidal self-directed violence. They received care in various health care settings, such as emergency rooms or units, day hospitals, stationary psychiatric departments of hospitals, or outpatient psychiatric settings. Data were collected through interviews in all studies [24-35] and additional observation in 2 studies [27,34]. Minors aged 11 to 17 years were interviewed in 3 studies [27,33,34]. Children aged 11 to 14 years were observed in one study, and their parents were interviewed [27]. HCPs were interviewed in 3 studies [24,34,35], and 2 interviewed family members [27,35]. A detailed overview of the studies in the meta-synthesis is provided in Table 1.

**Figure 2:**
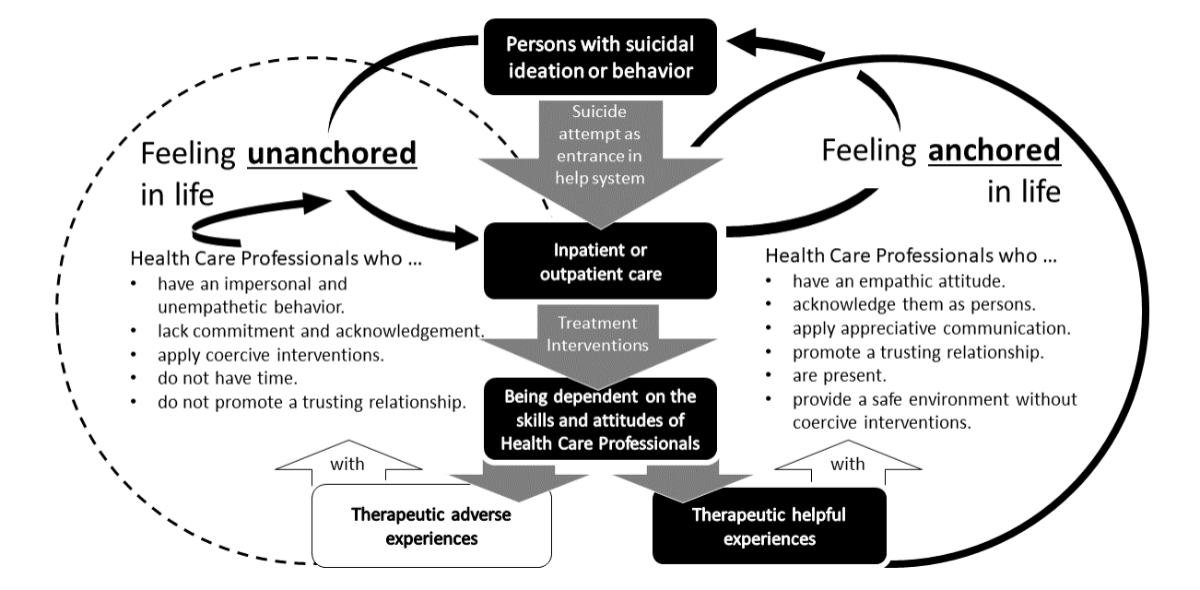
Conceptual model of therapeutic experiences.

In order to address the research question, a conceptual model was synthesized based on the analysis of all articles comprising two main concepts that reflect experiences during inpatient or outpatient care of people with suicidal ideation and behavior. The first main concept, “from suicidal ideation and behavior to feeling anchored in life,” describes the person with suicidal thoughts or behaviors in a process from feeling unanchored while experiencing a suicidal crisis to feeling anchored in life at the end of the subsequent recovery. This phenomenon includes the categories that describe the experience of suicidal ideation and behavior: (1) suicide as an option, (2) communication, and (3) transformation. Persons who ideate or attempt suicide are individually motivated to consider suicide because they cannot communicate their suffering. When speaking about suicide ideation is not taken seriously, they can experience suicide attempts as transformations. Moreover, suicide attempts serve as entrances to health care services. During the recovery process, individuals are being cared for by HCPs. The second main concept describes the “dependency on the skills and attitude of HCPs” in the recovery process. This phenomenon includes the categories (1) “adverse therapeutic experiences” and (2) “helpful therapeutic experiences” (2). These findings reveal certain hindering and helpful skills and attitudes of HCPs. Helpful skills support affected persons in reaching their feeling of being anchored in life again. Hindering skills are experienced negatively and might lead to prolonged or maintained feelings of being unanchored in life. Textbox 1 provides an overview of the identified concepts, categories, and subcategories. The conceptual model is presented in Figure 2. The model, its inherent concepts and categories, as well as the corresponding findings, will be presented subsequently.

**Table 1:** Overview of included studies.

Author(s), year	Country of study	Sample/setting	Focus of interest	Method/analysis
Berg, Rørtveit, Walby, & Aase, 2020 [29]	Norway	18 adults (mean age 40) with suicidal ideation and behavior in psychiatric wards in hospitals.	Exploring the experiences of persons in suicidal crisis with safe clinical practice during hospitalization.	Semistructured interviews; phenomenological-hermeneutic approach
Cardell & Pitula, 1999 [26]	USA	20 adults (mean age 32 years) with suicidal ideation in psychiatric wards in hospitals.	Exploring the experiences of persons with suicidal ideation who have been constantly monitored within the last 2 weeks to determine whether this protective intervention had therapeutic benefits for the affected persons.	In-depth interviews, at least 2 times for each person; grounded theory analysis (Hutchinson)
Cutcliffe, Stevenson, Jackson, & Smith, 2006 [31]	UK	20 adults who ideated or attempted suicide. They received care from “emergency” psychiatric services as inpatients, outpatients, or day hospital patients.	Investigating whether and how psychiatric or mental health nurses provide meaningful, caring care to persons with suicidal ideation or behavior.	Semistructured interviews; analysis adhered to principles of grounded theory (Glaser)
Cutcliffe, McKenna, Keeney, Stevenson, & Jordan, 2013 [30]	Northern Ireland	36 male persons with suicidal ideation or behavior between 18 and 34 years old who had been treated in mental health inpatient or outpatient facilities.	Developing a theory on how informal and formal services can be better configured or reconfigured to respond more effectively to the needs of young men with suicidal ideation or behavior.	Semistructured interviews; analysis based on principles of grounded theory (Glaser & Strauss)
Hagen, Knizek, & Hjelmeland, 2018 [32]	Norway	5 adults with suicidal ideation or behavior between 33 and 54 years old who had been admitted to a district psychiatric center.	Exploring the experiences of former suicidal inpatients with treatment and care in psychiatric wards.	Semistructured interviews; interpretative phenomenological analysis
Holliday & Vandermause, 2015 [33]	USA	6 adolescents (15-19 years) who were treated in an emergency room after a suicide attempt.	To gain a comprehensive understanding of the experiences of adolescents who attempted suicide and were taken to an emergency room and their meaning of ideating or attempting suicide as adolescents.	Open, unstructured interviews; Heideggerian hermeneutic methodological approach, phenomenology
Lees, Procter, & Fassett, 2014 [24]	Australia	9 adults (mean age 41 years) receiving care due to suicidal crisis and 11 mental health nurses who assisted persons in suicidal crises (hospital and community setting).	Exploring the experiences and needs of individuals who had a suicidal crisis, the degree to which their needs have been met, the role of mental health nurses, and the key factors to improve quality of care.	Qualitative findings from a multimethod study. In-depth, semistructured interviews; analysis based on critical discourse, constant comparison, and classical content analysis
Montreuil, Butler, Stachura, & Pugnaire Gros, 2015 [27]	Canada	5 children (11-14 years) with suicidal risk factors and one of their parents. They were recruited from pediatric mental health inpatient, outpatient, and day hospital settings.	Assessing perceptions of children with risk factors associated with suicide and their parents regarding helpful care in a pediatric psychiatric setting.	Observations of children and semistructured interviews with parents; inductive data analysis (Colaizzi)
Samuelsson, Wiklander, Asberg, & Saveman, 2000 [25]	Sweden	18 adults (18-53 years) who attempted suicide and were treated in an inpatient psychiatric ward.	Investigating the experiences of patients in a psychiatric ward after having attempted suicide.	Semistructured interviews; content analysis (Burnard)
Sun, Long, Boore, & Tsao, 2006 [34]	Taiwan	15 persons (16-47 years) with suicidal ideation or suicidal behavior and inpatient treatment on psychiatric wards; 15 psychiatric nurses	Exploring experiences of nurses and affected persons to develop a care theory that guides the care of people with suicidal thoughts or behavior.	Semistructured interviews and observations; grounded theory (Strauss & Corbin; Eaves)
Sun, Long, Tsao, & Huang, 2014 [35]	Taiwan	14 adults (22–83 years) who attempted suicide were recruited from an outpatient clinic; 6 caregivers (family members, psychiatrists, a psychiatric nurse)	Exploring contextual and intervening conditions that influence individual healing after a suicide attempt.	Semistructured interviews; grounded theory (Strauss & Corbin)
Vatne & Nåden, 2014 [28]	Norway	10 adults (21-52 years) who ideated or attempted suicide. They were recruited in emergency psychiatric units and from a crisis resolution team.	Exploring the experiences of being suicidal and encounters with health care personnel.	Semistructured interviews; thematic analysis (Braun & Clarke)

**Concepts, categories, and subcategories (excerpts from data analysis).**

**From suicidal ideation and behavior to feeling anchored in life:**

Suicide as an option:

- Being unanchored in life
- Wanting to escape
- Seeing no way out

Suicide as communication:

- Having difficulties speaking about suicidal ideations
- Hiding behind a mask
- Shouting without words

Suicide as transformation:

- Reconnecting through help
- Giving meaning to life
- Moving towards feeling anchored in life again

**Dependency on the skills and attitude of health care:**

Adverse therapeutic experiences:

- Having an impersonal or unempathetic attitude
- Lacking commitment and acknowledgment
- Applying coercive interventions
- Lacking time
- Not building a trusting relationship

Helpful therapeutic experiences:

- Empathetic attitude
- Acknowledging affected persons
- Appreciative communication
- Promotion of a trusting relationship
- Presenting and providing a safe environment

Legend:

**Concept:**

Category

- Subcategory 1
- Subcategory 2

### From Suicidal Ideation and Behavior to Feeling Anchored in Life

While having suicidal thoughts or attempting suicide, affected persons experience feelings of being unanchored in life. For different reasons, they consider “suicide as an option” and ideate or attempt suicide. The motivation for suicide has to be taken into account to understand the phenomenon of being unanchored in life. A suicidal crisis is multifactorial and is described individually [24]. For some persons who attempted suicide, the battle in their brain was a motive. They describe it as wanting to flee, having lost control of thoughts, and their mind playing tricks on them. The authors explain “that the thoughts flooded their brain making it difficult for them to ‘think in reality’ and that such thoughts enhanced the confusion and ambiguity” [33]. At the same time, participants describe having low self-esteem [32].

Another motive is the desire to end or escape from emotional as well as psychological pain and suffering. Painful experiences and the inability to cope with life or problems were further statements [24,33,34]. An interviewee felt ”better off dead” [24]. Feelings and thoughts of powerlessness, loneliness, inability to solve problems in life, manipulation of others, or feeling down led to considering or attempting suicide as a way out [24,33,34]. Others stated mourning the loss of an important fellow human being was a motivation for suicide [24,28]. For interviewed persons from Northern Ireland, being male was one reason for suicidal ideation, as society expects great and strong men not to show their feelings and problems [30]. Some of those persons who ideated or attempted suicide reported multiple strains in everyday life. Various physical and psychological problems or illnesses, stress, interpersonal stress, social exclusion, and unpredictable life events also contribute to considering or attempting suicide [24,28,30,34].

Another subcategory describes “suicide as communication.” Persons attempting suicide described that they wanted to hide their suffering and end their indescribable suffering. The suicide attempt was a cry “for” help but not only in the sense of calling attention. It was like a cry “of” pain that allowed their pain to become visible to others. Before the suicide attempt, they could not communicate their suffering to their environment through speech. The suicide thus served as a message for others. They put their pain into a social context by making others “hear” their pain [28,33]. Persons with suicidal behavior did not consider themselves ill but realized their problems and knew they needed help [25]. However, it seemed difficult for them to ask for help and access health services. They point out that they were in a vulnerable situation. They express shame for ideating and attempting suicide and the fear of forced admission [25,27,28]. They could talk about physical suffering but not about their sadness, hopelessness, suffering, and feelings. When someone talked about suicidal thoughts, that person seemed weak [28,33]. They feared being judged and stigmatized [30]. They told no one about their suicidal thoughts and wore a mask that concealed their true feelings. Suicide is still a taboo subject, and they do not want to be condemned by others. The thoughts of suicide are perceived as unusual and incomprehensible by the people around them, sometimes leading to the humiliation of the persons with suicidal self-directed violence. Thus, many of those affected hoped that someone would notice their intentions and make small gestures without verbally expressing themselves [28,29,33]. Others tried to communicate but still required the HCPs to interpret their words and understand the severity of their suicidal ideations [29].

The subcategory “suicide as transformation explains” the process of feeling unanchored during suicidal ideation and attempting suicide to recovery with professional help and feeling anchored again. Before the suicide attempt, young people describe the feeling of being disconnected. They feel lonely, rejected as outsiders, neglected, unloved, and spiraling down. They believe that no one else could understand these feelings of emotional pain [33]. Others have the feeling of “falling between two stools” and are in a struggle for life in which support from HCPs is needed [28]. One interviewee described it as follows: “You feel like you’re in the bottom of a hole and its all black and you don’t’ see nothing…you’re just trapped” [33].

Affected persons state that without the suicide attempt, they would not have received the necessary treatment. Some consider attempting suicide when talking about suicidal ideation is not taken seriously. Although some already had treatment in the past, they described the treatment after attempting suicide as helpful and gaining better insights [25,28,33]. Consequently, the attempt can be the entrance to receiving more appropriate support from health care services. Persons with suicidal ideation or self-directed violence are given access to treatment, similar to the beginning of the transformation from a death wish to a wish to live. However, the process was perceived as challenging [28,31,33,35]. Hagen et al [32] state that affected persons are in the process of development that relates to existential, relational, and practical aspects of life: “The experiences appeared to be a part of a personal recovery process that was influenced by the support they received form [sic] mental health workers.”

Cutcliffe et al [31] describe the change from death orientation to life orientation in a three-stage healing process that reconnects the person with humanity. The first stage is “reflecting an image of humanity” [31]. Those affected describe that they restore confidence in humanity through reconnecting with HCPs who function as representatives. They can give persons with suicidal ideation or behavior the feeling they are not being left alone and that they care for them, show compassion, and try to offer them help [31,34]. People with suicidal ideation or behavior need to have someone who listens and shows understanding and interest [31,33,34]. Persons with suicidal thoughts or behavior describe that HCPs can show them they are important and an individual. The relationship with HCPs is different from that with family and friends, which gives them a feeling of emancipation [31]. Most interviewed persons reported that their hospitalization was helpful. An affected person may assume he or she has ruined other persons’ confidence in oneself by attempting suicide, which leads to the feeling that he or she needs ”some kind of babysitter,” which means inpatient observation and care [25]. Furthermore, inpatient care helps persons who attempt suicide realize that connecting with others can help in recovery, and they still have a connection to their support group. It also helps to reflect on one’s constructs influenced by their hopelessness [31,33]. Affected persons need to be treated without judgment and experience acknowledgment of their narratives and acceptance from others and themselves [30,31,33,35].

In the second stage, “guiding the individual back to humanity,” nurses have a more active part in their nursing role than in the first stage [31]. They support the individual in taking on new perspectives to think about one’s personality, life, and opportunities [31]. Individualized treatment and care enable affected persons to disclose their thoughts and increase their self-awareness [32]. They experience a transformation of their understanding “from feeling uncared for to understanding that others have always cared for them” [33]. Persons with suicidal ideation or behavior realize that many people have similar emotional problems. They learn to accept help and have the ability to accept support, reconnect, and form new connections. They experience and accept support from the family by reconnecting with them. Those affected find more harmony with society again [30-33,35]. The inclusion of strong social support systems served as a helpful therapy. This reduced stress and made it possible to overcome difficulties. The awareness helped them realize that their families did not reject them because of their suicidal nature and instead forgives them; their families will continue to care for them, accept them, and help them in their therapy [30,35]. During the recovery process, they want to live in a friendly environment and experience being needed by others. This helped them feel valuable and accepted, enabling self-acceptance [35]. Being accepted by significant others promotes increased hope for persons with suicidal ideation or behavior [30].

In the third stage, “learning to live,” persons with suicidal ideation or behavior focus on the balance between reconnecting with family, friends, and other people and disconnecting gradually from HCPs [31,32]. They successively learn to give meaning to life again, to make plans, and to set goals. They make sense of the events and gain a new understanding. Giving meaning to life and learning to live is crucial for the recovery process [30,31,33]. It is like becoming a new person and building oneself up again [30,33]. It is important for those affected to feel needed and accepted in society again [35]. Nevertheless, some people express fear of coming back to reality and feel the potential disappointment of others [33]. Feeling able to cope with symptoms and life situations is a prerequisite for discharge from the hospital [29]. Psychiatric treatment can give persons with suicidal ideation or behavior the opportunity to reflect on themselves and their lives. The journey to recovery is a long process, but it provides the opportunity to connect with other people, gain access to the social network, and establish trust [30]. The predictability of follow-up treatment after hospitalization ensures safety. In contrast, feeling unprepared can trigger a suicide attempt, as one participant notes [29].

Affected persons can re-establish internal emotional control when treated as individuals as their stressors can be addressed [29]. Through professional care, persons with suicidal ideation and self-directed violence can reach the feeling of being anchored in life again. Many realize they need support from HCPs. Professional care is central to this recovery process. Experiences during inpatient or outpatient care affect the recovery of persons with suicidal ideation or behavior [25,30,31,33,34]. These experiences with professional care are described in the second concept that follows.

### Being Dependent on HCPs’ Skills and Attitudes

The second concept describes experiences of persons with suicidal thoughts or behavior as dependent on the HCPs’ skills and attitudes. During inpatient or outpatient care, affected persons need professional care and support. According to the HCPs’ skills and attitudes, the analysis revealed that affected persons experienced therapeutically adverse or helpful aspects during their recovery process.

### Therapeutically Adverse Experiences

Persons with suicidal ideation or behavior experienced these skills and attitudes as therapeutically hindering (ie, having an impersonal or unempathetic attitude, lacking commitment and acknowledgment, applying coercive interventions, lacking time, and not building a trusting relationship).

Impersonal HCPs and those who lack empathy and commitment are experienced negatively by those who ideated or attempted suicide [25,26]. It is perceived as hindering when HCPs have judgmental attitudes, especially when combined with the affected persons’ negative feelings regarding the provided care [34]. A lack of commitment is described as therapeutically adverse. Some HCPs seem to be simply doing their job, prioritizing their needs without using the available time effectively or supporting persons with suicidal ideation in the struggle for their lives [24,25]. A lack of commitment and emotional confirmation also results in persons with suicidal behavior not wanting to talk about themselves [25,29]. HCPs who act impersonally or do not show empathy can contribute to affected persons’ damaged self-esteem, increased anxiety, and dysphoria [26]. When affected persons arrive in the emergency room, they experience a lack of consideration [25]. For example, after a suicide attempt in the emergency room, the physical, but not the psychological well-being, was assessed, with the affected person noting, “they just checked my heart and said everything was fine” [33]. They describe HCPs as if they were distracted, disinterested, indifferent, or uncaring. They have the impression that no one seems to care for them and as if they were a burden [25,26].

Affected persons and HCPs state a lack of knowledge [25,28,32,34].“Of particular concern was the finding that many nurses did not have the best possible attitude, education, training, or support to optimally meet the challenges and opportunities at hand, and more fully realize therapeutic engagement” [24].

These attitudes towards care resulted in people feeling lonely and not cared for, which ultimately led to fear, aggression, and a lack of trust in HCPs, although they longed for trust [24,25,28]. Some people did not feel taken seriously in their illness or received inadequate responses and information. Without their perspective being acknowledged, they sometimes feel misunderstood [25,26,28,32,34]. “When the patients did not feel that they were confirmed, it sometimes gave rise to feelings of being burdensome, a desire to go home, or even another suicide attempt” [25].

Experiencing a lack of trust leads to withdrawal from seeking help from HCPs [29,32]. Coercive interventions are unhelpful because they restrict autonomy and privacy and lead to feelings of confinement [24,26,34]. According to the person who constantly observes them, persons with suicidal ideation have uncomfortable or distressing feelings. They prefer HCPs to be supportive rather than impersonal and detached. An example of this behavior is “not responding to the participant’s initiation of conversation and perceived hostile facial expressions” [26]. In the absence of engagement, affected persons experience interpersonal isolation, distress, objectification, and loss of control [24]. They sometimes feel treated like a child, which was experienced as humiliating [25,28,34]. Persons with suicidal ideation or suicidal self-directed violence describe environmental stressors as obstructive to their therapeutic experience. For example, television noise was described as annoying and caused stress and excessive demands, as participants had to stay in recreation rooms [26]. Others perceive a lack of respect when HCPs behave like guardians [25]. In situations with coercive interventions, communication is seen as very important. If HCPs failed to communicate relevant information (eg, about constant observation and the observer, or did it abruptly), affected persons stated frustration, irritability, and anxiety [26].

Some people mentioned the lack of time for care, with busy HCPs looking after many patients and having no time for the affected persons [24,32,34]. Additional adverse contextual factors included the lack of teamwork and support and inadequate professional supervision [24]. Some HCPs “did not prioritize interpersonal engagement, or thought it was sometimes inappropriate or countertherapeutic” [24]. Affected persons wanted more time for conversations to establish a close therapeutic relationship. They longed for dialogue and trust [28]. Without individual treatment, they experienced that they were a risk to themselves after discharge [29].

This also had an impact on the relationship between persons with suicidal behavior and HCPs. Instead of sharing their thoughts, they kept them to themselves if they did not feel safe with the HCP [25,28,32]. It takes time to establish a close relationship. However, sometimes this relationship was not possible due to “bad chemistry” [28]. Affected persons state a lack of therapeutic engagement with little interaction and few possibilities to talk [24]. Others experienced HCPs changing topics rather than talking about themes that persons having attempted suicide need to discuss. This leads to feelings of humiliation and annoyance [28].

### Therapeutically Helpful Experiences

In addition to therapeutically adverse experiences, persons with suicidal thoughts or behavior also experience therapeutically helpful experiences. These concerns relate to an empathetic attitude, acknowledging affected persons, appreciative communication, promoting a trusting relationship, and presenting and providing a safe environment.

Persons with suicidal thoughts or behavior in the included studies describe the importance of an empathetic attitude from HCPs [24-26,34]. HCPs should be warm, pleasant, talking, listening, and understanding [27]. For those affected, it is important that HCPs honestly put themselves in their position and show concern and care [24,28,31]. They need HCPs “to be willing and able to listen intently […], to attempt to see the world ‘through the eyes of the young person and be supportive” [30]. Therefore, being nonjudgmental is important [30,31,34]. HCPs with knowledge, experience, and understanding of physiological processes and stages in mental illness are seen as helpful [27,28,32]. An optimistic attitude on the part of the HCPs also provided therapeutically helpful care and increased self-esteem. The optimistic behavior of care is perceived as loving, helpful, and hopeful [26]. It is essential for those affected to be regarded as individuals, supported in their autonomy, and not condemned for their illness. This enabled them to realize that they were worth something and that they were being cared for [24,25,29,34]. Persons with suicidal ideation and behavior need acknowledgment of their person and their illness [26,35]. It was also experienced as helpful when professional care and treatment were individualized to the needs of the affected persons [27,29,32]. Persons with suicidal ideation and behavior experienced safe clinical practice when they received tailor-made treatment. This type of treatment relieved their emotional pressure as stressors were addressed [29].

Communication between HCPs and those affected plays a central role in recovery. The basis is a holistic assessment of persons with suicidal thoughts or behavior, including their needs and causes of suicidal tendencies. Some people actively seek dialogue with HCPs to talk about their suicidal thoughts and behaviors, about issues they have not yet entrusted to anyone, or answers to questions they have [24-26,28]. They value cooperation and open communication between different HCPs to understand themselves and plan their treatment [27,34]. Talking to HCPs and being understood and supported was felt by some to be vital, although talking about it was initially felt by many to be terribly painful [24,25,31]. In addition, speaking, active listening, and being taken seriously are important. The commitment of HCPs is essential to explore the complexities of suicidal ideation in affected persons [24,28,29,32]. Discussions between those affected and HCPs without judgment, and on the same level, helped many sort out their thoughts [24,25,28,31]. One interviewee put it as follows:

She wasn’t...yes, “pitying” again then. We were two people talking together on equal terms, not prisoner and jailer...She would not divert the conversation, no matter what

28

It was considered important that HCPs should not scream but speak in a calm tone [27]. The direct inquiry into suicidal thoughts or plans was also positively perceived by those affected [28,34]. Some people found the denial of these thoughts impossible because HCPs often knew persons with suicidal self-directed violence well and recognized their needs and feelings through nonverbal communication [25,29].

A trusting relationship between the HCPs and their entrusted persons with suicidal thoughts or behavior served as a basis for both parties [24,25,34]. Participants noted that having an open conversation requires a “good chemistry” and connection with a health care professional [32]. Many persons with suicidal thoughts or behavior had difficulties trusting people and had no support from friends or family, so trusting caregivers was important [28,31]. A trusting relationship at the same level between the HCPs and those affected had a positive effect on suicidal tendencies [28,29]. It is essential for the therapeutic relationship that HCPs show appreciative behavior, respect, and interest for the well-being of persons with suicidal thoughts [26,32]. It is important that HCPs introduce themselves with their name when meeting each other the first time [26], and they should also know the affected persons’ names [27]. Developing a caring relationship can be promoted through getting to know each other and personalized care [27]. Persons with suicidal self-directed violence want to be met on equal terms, which “is a situation whereby the parties accept each other’s inherent value” [28]. The therapeutic relationship can be established with communication basics, such as showing compassion, acceptance, and appreciation and serving the healing process.

Additionally, worthiness was fundamental as it increased well-being and reduced anxiety, dysphoria, and loneliness in persons with suicidal ideation [26]. A sense of companionship provides a sense of safety and well-being and enables affected persons to disclose suicidal thoughts [32]. Therapeutic closeness served as the basis for subsequent interventions [30,32]. Promoting trust with HCPs helped the affected person reconnect with an individual as the first step before reconnecting with their social environment [31].

Another helpful experience with HCPs was present [24,26,27,29,35]. Although it sometimes felt difficult, especially for children and adolescents, to ask for help, it was important to persons with suicidal self-directed violence that someone was always available to give them security and help. The HCPs were described as very sensitive and often recognized how they felt, whether they needed time to talk or wanted to be left alone [25,27,35]. Some of the affected persons also used HCPs after discharge and called them by telephone [25,27]. Many of them describe that the greatest help was knowing that care was always present and that someone was available to take time for them [24,25,29]. Parents of minors with suicidal ideation also described this as very helpful and used the opportunity to call at their convenience [27].

Being present also affected the therapeutic environment. Experiencing protection and safety through 24-hour care of HCPs positively affects therapy and the relationship between caregivers and persons with suicidal ideation or behavior [27,29,34,35]. Protection and security within the institution have been described by many as a reason to stay, live, and take time for therapeutic interventions in this environment. However, it was not easy for many of those affected to be treated in a psychiatric institution [25,26,35]. However, many expressed that the institution would provide them with personal security, protection, peace, and no stress [29,35]. Due to the vigilant presence, constant observation, and physical presence of HCPs, suicide was hardly possible. This is also due to locked stations and the prohibition of bringing sharp objects such as knives and weapons. As a result, some persons with suicidal thoughts or behavior felt safer from their suicidal impulses thanks to the institution [26,29,34]. Additionally, a quiet, friendly atmosphere, a good climate on the ward, and a meeting on the same level had a positive effect on the therapeutic environment [28,35].

Persons with suicidal self-directed violence also experienced care without coercion as therapeutically helpful. The participants expressed that they felt relief through unconstrained hospitalization. Although hospitalization was described as a terrible feeling, HCPs stressed that the need for inpatient care was helpful if treated voluntarily. In addition, not being forced to speak but simply to be allowed to sit quietly was experienced positively [25,28].

## Discussion

### Principal Results

This study aimed to synthesize qualitative research to develop a model that supports HCPs in their work with persons with suicidal thoughts or behavior and provides an evidence base for developing eHealth tools. The results of the meta-synthesis revealed two central categories shown in the conceptual model (Figure 2). The first concept describes persons with suicidal ideation or behavior in their struggle as they feel unanchored in life to feeling anchored in life again. A suicide attempt can function as an entrance to health care services to more easily achieve feelings of being anchored in life again. When receiving inpatient or outpatient care by HCPs, the affected persons are dependent on the skills and attitudes of the professionals present. Helpful therapeutic experiences stated by persons with suicidal ideation or behavior have been synthesized. HCPs’ helpful skills and attitudes support persons ideating or attempting suicide to feel anchored in life again. On the other hand, the identified therapeutically adverse skills and attitudes from HCPs may hinder affected persons in proceeding with their recovery and allow them to maintain the feeling of being unanchored in life.

In the following discussion, we focus on two main aspects. First, a suicide attempt can function as an entrance to health care services. Second, adverse therapeutic experiences that hinder affected persons from feeling anchored in life again versus helpful experiences that promote their way toward being anchored. Aspects concerning eHealth are taken into account within each point.

### Need for More eHealth Devices to Prevent Suicide Attempts as Entrances to Health Care Services

The meta-synthesis revealed that persons with suicidal ideation sometimes considered attempting suicide when they felt they were not being taken seriously. Through suicide attempts, they enter the health care system and receive necessary or more appropriate treatment [25,28,33]. Our results and qualitative analysis of motives show that attempting suicide is not a conscious decision but rather a complex interaction of different factors [37]. Another qualitative study focused on experiences disclosing suicidal thoughts. Persons who attempted suicide experienced difficulties disclosing primarily during and after the crisis. They did not find the right words, found themselves unable to share their thoughts, or feared being stigmatized by family, friends, or HCPs [38]. They felt ambiguous about sharing and being unable or fearing to do so. Nevertheless, one interviewee stated, “Nobody hears a silent cry for help” [38]. Difficulties in disclosing, or feeling one is not being taken seriously, can result in an initial or subsequent suicide attempt, which can be the first step in a recovery process as most unsuccessful suicide attempts gain access to appropriate health care services. Experiencing helpful skills and attitudes from HCPs is critical to feeling anchored in life again. Professional care plays a central role in helping individuals find their way back to feeling anchored in life again. The phenomenon identified in our meta-synthesis of attempting suicide as an entrance to health care services has not been found in the literature. The reason could be that we identified it from our analysis of persons who attempted suicide and reflected on their experiences. Therefore, it may not be an intention of persons with suicidal ideation but may be seen as such when reflecting it from a future perspective.

Regarding the aims of this study, using eHealth tools could potentially intervene when persons have suicidal thoughts or behavior or are at risk for self-harm [14,15,39]. Positive outcomes of utilizing eHealth applications included reducing depression, psychological distress, and self-harm [14], while others reduced suicidal ideation [15,39]. Using an app is an easy and anonymous way to deal with upcoming thoughts. Other possibilities are web-based technologies or social networks. In particular, younger people can be more easily addressed through technology-based interventions for suicide prevention [40]. A study stated that young people confronted with depression, suicidal ideation, or stress are less likely to talk to their parents about their problems and more likely to speak to no one [41]. It is challenging to identify people at risk in order to manage the crisis with close persons. Holland et al [19] propose the presence of an adult as a backup to ensure safety during calls and video sessions. A significant improvement for quality and outcome of intervention has also been found with the involvement of family or caregivers as youth and family receive coping tools and psychoeducation. One opportunity would be to detect people at risk in social networks and facilitate access to a supportive network and specialists. However, there is a lack of studies concerning suicide prevention in social networks [40]. Young persons with suicidal ideation used four types of technology-based telemental health resources: self-help, anonymous chat, crisis text lines, and online therapists or counselors [41]. Online interventions can be helpful, especially if persons with suicidal ideation or behavior have difficulties disclosing their crisis to family or friends, as studies have shown [38,41].

Nevertheless, it is vital to guide affected persons to helpful resources. A survey indicated that a search for suicide-specific themes could lead to preventive (68%) resources but also to mixed (22%) and neutral (8%) content concerning attitudes towards suicide or even prosuicide content (1%) [42]. There is a growing body of interventions for suicide prevention. In their systematic reviews, Melia et al [14] and Kreuze et al [15] conclude that there are technology-based interventions for suicide prevention. However, further research is needed to evaluate their efficacy. Difficulties also arise in the missing evaluation of self-management applications for suicidal thoughts or behavior [39,40].

### Need for Integration of HCPs’ Helpful Skills and Attitudes in eHealth Applications

The meta-synthesis revealed that persons with suicidal thoughts or behavior had therapeutically helpful experiences when HCPs had an empathetic attitude, acknowledged affected persons, used appreciative communication, were present, promoted a trusting relationship, and provided a safe environment. Two systematic literature reviews, a meta-synthesis, and a mixed-methods study of persons who self-harm and have suicidal ideation or behavior identified a positive relationship between patients and HCPs as crucial. They find it important that HCPs are supportive, compassionate, and ready to listen [43-46]. Although 2 of these studies are about self-harm, the focus on the importance of the relationship is the same as that shown in our results. Understanding and nonjudgmental HCPs were seen as important for future help-seeking. Studies also state the need for boundaries through a safe environment and that sometimes safety measures as special observations are appropriate [43,45,46]. This is congruent with our result of HCPs providing a secure environment. Knowing HCPs are present creates a feeling of comfort for persons with self-harm, suicidal ideation, and behavior [44-46].

Our results show that affected persons experienced skills and attitudes of HCPs as therapeutically adverse when they have an impersonal, unempathetic attitude, lack commitment and acknowledgment, apply coercive interventions, lack time, and do not promote a trusting relationship. Similar results were found in literature about adults who self-harm, have suicidal ideation, or behavior. HCPs who have a judgmental attitude, are unsupportive, lack empathy, or exert power are seen as part of unsatisfactory care [43-46]. Negative experiences could even be a barrier for future help-seeking, while trust in services encouraged future help-seeking [43,44]. Hagen et al [8] examined therapist challenges with persons who self-harm and have suicidal thoughts or behavior. The interviewed psychiatrists and psychologists described challenges between categorizing the illness and connecting with the affected persons while following guidelines for diagnoses and treatment. Moreover, they experience challenges forming a trusting relationship due to limited time in direct care and formal obligations. Consequently, therapists spend less time with affected persons, and the greater part of direct care is done by other staff members such as psychiatric nurses. This underlines the importance of cultivating helpful skills and attitudes in nurses.

For eHealth interventions, it is a challenge to consider how affected persons benefit from the skills and attitudes of HCPs. Some eHealth resources are solely informational or unguided self-management applications, while others provide exchanges with other persons seeking help or provide virtual contact with HCPs. From the meta-synthesis results, it has to be considered how an empathetic attitude, appreciative communication, being present, and promoting a trusting relationship can be transferred into an eHealth application. The use of artificial intelligence for mental health care provides a multifaceted opportunity, although unanswered ethical questions remain [47]. A literature review identified chatbots that can be used in mental health. They are applied for different purposes, such as therapy, self-management, counseling, or diagnosis. For example, one was identified for screening symptoms of depression and suicide [48]. These chatbots could be “trained” with helpful skills and attitudes. In addition to unguided self-management applications, the focus should be on those with direct contact and crisis support. Via video conferencing, telemental, or similar tools, affected persons can communicate with HCPs directly. During the COVID-19 pandemic, a survey reported mainly positive experiences of users with telepsychiatry. Most of the participants (82.2%) found that the overall experience with telepsychiatry was excellent or good. In addition, participants either agreed or strongly agreed (63.6%) that the remote sessions were as helpful as in-person treatment [49]. Another opportunity could be a brief text message-based intervention that showed the potential to support persons who attempted suicide in connecting them with a crisis support team to reduce re-attempts [50]. Franco et al [40] stated an upward trend in using technology-based interventions for suicide prevention. However, these are mostly in English [40], presenting a barrier for persons who are not familiar with the English language.

### Strengths and Limitations

The strength of this meta-synthesis is the conceptual model derived from how persons with suicidal ideation and behavior experience inpatient and outpatient care. To our knowledge, this is the first meta-synthesis of these experiences with professional care. This is important for ethical reasons, as no affected persons need to be newly involved in this meta-synthesis. The results can be used as a basic needs assessment for eHealth development and nurture an empathetic culture among HCPs. However, our findings are limited by the different study designs exploring the experiences of affected persons. Half of the studies relied on grounded theory or were analyzed with the principles of grounded theory. The other studies used content analysis, inductive data analysis, or a Heideggerian hermeneutic approach. Through the grounded theory–based analysis, we gained a high theoretical level of abstraction. Nevertheless, we could not formulate theory but could speak of a conceptual model. Including experiences from the different samples broadened the range of perspectives. However, it must be taken into account that the samples had different cultural origins. Therefore, we suppose the results could be transferred to different cultural contexts, but they must be checked beforehand.

### Conclusions

We derived a conceptual model of experiences made by persons with suicidal ideation and behavior. The model showed the main helpful skills and attitudes of HCPs that can support affected persons to be anchored in life again. Conversely, we also identified hindering skills and attitudes that lead to adverse therapeutic experiences, which may prolong the recovery of persons with suicidal ideation and behavior.

We focused our research on persons who have suicidal ideation or have already attempted suicide. The discussion in previous studies showed that persons with self-harming behavior experience similar challenges and can likewise benefit from eHealth tools that address suicidal thoughts or behavior. It is useful to address these groups with one application, as the boundaries may be indistinct for affected persons.

This meta-synthesis has some practical and theoretical implications. As practical implications, the results can be used as a blueprint for technicians and HCPs to develop eHealth interventions. These could especially address younger persons, as they are more likely to use online resources or eHealth applications in cases of suicidal ideation or behavior. Especially during the COVID-19 pandemic, eHealth tools are a convenient solution. Another practical implication addresses HCPs. Our results show that suicidal ideation should be taken seriously by HCPs. They could use the conceptual model for training and education to improve professional care and improve outcomes for affected persons. HCPs need to be sensitized for the effects their skills and attitudes have on persons with suicidal ideation and suicidal self-directed violence. They should react with appreciative communication and an empathetic attitude and be present to promote a trusting relationship. Moreover, they should ensure a safe environment to help affected persons feel anchored in life again without using a suicide attempt as another effort to benefit from health care services.

As theoretical implications, further research is needed. Research should focus on experiences made by persons with suicidal ideation or behavior from a hermeneutical perspective. The meta-synthesis with the derived conceptual model can function as a basis for developing new interventions to support affected persons. These interventions could focus on deepening the helpful skills and attitudes of HCPs in interactions with persons with suicidal thoughts or behavior. Other interventions should promote eHealth applications for affected persons, which are evaluated as to whether they accurately fit and support persons with suicidal thoughts and behavior. More research is also needed to identify helpful interventions for affected persons. Moreover, a questionnaire could be developed from the conceptual model to promote the quality of care of affected persons.

## Abbreviations

CASP: critical appraisal skills program
HCP: health care professionals
